# The role of ZC3H13 in promoting M2 macrophage infiltration via m6A methylation in esophageal squamous cell carcinoma tumor progression

**DOI:** 10.3389/fimmu.2025.1612041

**Published:** 2025-09-01

**Authors:** Qihang Yan, Chendi Xu, Li Gong, Dachuan Liang, Jie Yang, Yuzhen Zheng, Junye Wang

**Affiliations:** ^1^ State Key Laboratory of Oncology in South China, Guangdong Provincial Clinical Research Center for Cancer, Sun Yat-Sen University Cancer Center, Guangzhou, China; ^2^ Guangdong Esophageal Cancer Institute, Sun Yat-Sen University Cancer Center, Guangzhou, China; ^3^ Department of Thoracic Surgery, The Sixth Affiliated Hospital, Sun Yat-Sen University, Guangzhou, China; ^4^ Biomedical Innovation Center, The Sixth Affiliated Hospital, Sun Yat-sen University, Guangzhou, China

**Keywords:** esophageal squamous cell carcinoma (ESCC), M2 macrophage, m6A, tumor microenvironment (TME), ZC3H13

## Abstract

**Introduction:**

ZC3H13 (zinc finger CCCH-type containing 13) is a member of the zinc finger protein family with regulatory roles in gene expression and represents a crucial m6A methyltransferase. However, the precise function of ZC3H13 in the esophageal squamous cell carcinoma tumor microenvironment (TME) remains incompletely understood. Our study primarily investigated the impact of ZC3H13 on m6A methylation modification in ESCC and explored the roles of ZC3H13 and M2 macrophages in ESCC.

**Methods:**

We employed bioinformatics analysis to assess the function of ZC3H13 in ESCC. Quantification of ZC3H13, CCL5, CXCL8, and macrophage infiltration in clinical samples and cell line-derived xenograft (CDX) tumor models was conducted using real-time quantitative PCR (qRT-PCR), western blot (WB), immunohistochemistry (IHC), Immunofluorescence (IF), and Enzyme-linked immunosorbent assay (ELISA). The colorimetric method was utilized to detect m6A methylation in cells and tissues. Tumor proliferation, migration, and invasion were evaluated using CCK8, EdU staining, colony formation tests, transwell assays, and CDX models.

**Results:**

We found that elevated ZC3H13 expression was positively correlated with m6A methylation modification in ESCC tumor tissue. ZC3H13 mutation led to abnormal nuclear metastasis of METTL14 and METTL3. Silencing ZC3H13 inhibited ESCC tumor growth and M2 macrophage infiltration in mice. ZC3H13 silencing also suppressed the expression of CCL5 and CXCL8 mRNA. M6A modification enhanced the stability of CXCL8 mRNA. ESCC tumors promoted the polarization of M0-M2 macrophages through the CXCL8-CXCR2 axis, which CXCR2 inhibitors or anti-CXCL8 antibodies could inhibit. Migration of M0 macrophages was facilitated by CCL5.

**Discussion:**

Our findings elucidate the connection between ZC3H13-mediated m6A modification and M2 macrophage infiltration in the ESCC-TME, resulting in M2 macrophage polarization and increased M2 macrophage infiltration.

## 1 Introduction

Esophageal cancer (EC) stands as one of the most prevalent gastrointestinal malignancies worldwide, ranking ninth in incidence and sixth in death rate among all cancers (1). The severity of this disease and its impact are emphasized by the annual toll of over 500,000 fatalities (2). Histopathologically, EC is primarily classified into esophageal squamous cell carcinoma (ESCC) and esophageal adenocarcinoma (EAC), each exhibiting distinct clinical features (3). EAC is predominantly observed in Western countries, while ESCC prevails in East Asian nations, particularly China and Japan (4). This geographical discrepancy suggests potential associations with race, climate, and lifestyle. Additionally, factors such as gender, age, excessive alcohol consumption, poor diet, and smoking contribute to EC risk (5). However, the molecular mechanisms underlying EC development remain unclear.

Current evidence suggests that m6A modification, the most prevalent RNA modification found extensively in eukaryotic cells, serves a vital function in gene expression control. Its involvement spans various cellular processes, including RNA processing, degradation, nucleoplasmic transport, and translation (6). A growing body of research indicates that m6A modification is closely linked to tumor initiation and progression. Its modification process mediates key aspects of tumor biology, including growth, invasion, metastasis, and resistance to chemoradiotherapy (7).

Within cells, m6A modification is primarily orchestrated by “writers”, “erasers”, and “readers” (8). The term “writers” refers to methyltransferases, encompassing METTL3, METTL14, WTAP, RBM15, ZC3H13, and KIAA1429 (VIRMA) (9). In recent years, the focus of studies on “writers” has predominantly centered on METTL3 and METTL14, with limited exploration of WTAP, RBM15, ZC3H13, and KIAA1429. Nevertheless, the roles of WTAP, RBM15, ZC3H13, and KIAA1429 remain integral to m6A modification. For instance, WTAP facilitates the m6A modification of lncRNA DIAPH1-AS1 in nasopharyngeal carcinoma (NPC), thereby increasing its stability (10). It has been reported that in osteosarcoma (OS), the highly expressed Circ-CTNNB1 interacts with RBM15 to stabilize its activity. This interaction promotes the expression of key glycolytic enzymes (hexokinase 2, glucose-6-phosphate isomerase, and phosphoglycerate kinase 1), consequently upregulating cell glycolysis and contributing to the progression of OS (11). In non-small cell lung cancer (NSCLC), VIRMA is highly expressed and associated with patient prognosis. VIRMA mediates the post-transcriptional repression of death-associated protein kinase 3 (DAPK3) through m6A modification. The downregulation of DAPK3 was found to promote cell proliferation and tumor growth (12).

Similarly, ZC3H13 assumes a crucial role in tumorigenesis. ZC3H13 mediates m6A modification of CENPK mRNA, which, in turn, binds to SOX6, promoting the nuclear translocation of β-catenin. This activation subsequently triggers the Wnt/β-catenin signaling cascade, enhancing cisplatin/carboplatin resistance and facilitating the epithelial-mesenchymal transition (EMT) process in cervical cancer (13). In contrast, ZC3H13 expression is downregulated in hepatocellular carcinoma (HCC). ZC3H13 downregulates PKM2 mRNA stability through m6A modification, thereby attenuating metabolic reprogramming, inhibiting HCC proliferation, and heightening sensitivity to cisplatin (14). Furthermore, reduced expression of ZC3H13 impedes the proliferation of laryngeal squamous cell carcinoma (LSCC) cells. ZC3H13 inhibits the DUOX1 gene via m6A modification, where DUOX1, in turn, suppresses the expression of GPX4 and F1H1, associated with ferroptosis, thereby down-regulating intracellular iron content in LSCC cells (15). Similarly, in papillary thyroid carcinoma (PTC), ZC3H13 downregulates its expression. By mediating the m6A modification of IQGAP1 mRNA, ZC3H13 diminishes IQGAP1 mRNA expression, consequently inhibiting PTC proliferation, migration, and invasion (16).

Given these diverse roles of ZC3H13 in regulating tumorigenesis through m6A-dependent pathways, it is reasonable to speculate that ZC3H13 may also influence esophageal squamous cell carcinoma (ESCC), particularly through modulation of the tumor immune microenvironment. However, the function of ZC3H13 in ESCC and its impact on tumor-associated macrophages remain poorly understood. Building on the evidence that m6A modification shapes tumor progression and immune evasion, we aimed to investigate the role of ZC3H13 in ESCC, with a specific focus on its regulatory effects on chemokines and macrophage polarization. Our study reveals a connection between ZC3H13, m6A modification, and immune remodeling in ESCC, providing novel insights into the molecular mechanisms driving ESCC progression and highlighting potential therapeutic targets.

## 2 Materials and methods

### 2.1 Tissue samples

Tumor tissues and adjacent normal tissues from 40 individuals with ESCC were sourced from Sun Yat-sen University Cancer Center. Patients underwent surgical treatment without prior antineoplastic therapy. All patients provided written consent, and the study received approval from the ethics committee of Sun Yat-sen University Cancer Center (SL-B2023-297-01).

### 2.2 Cell culture

In this study, various cell lines were utilized, including ESCC cell lines KYSE-30, KYSE-180, KYSE-150, KYSE-410, and KYSE-510, along with TE-13 and TE-7. Additionally, the viral packaging cell line HEK293-FT, human esophageal epithelial cell HET-1A, and human monocytic leukemia cell line THP-1 were employed. These cell lines were deposited at the Guangdong Esophageal Cancer Institute. The procurement details of specific cell lines are as follows: KYSE-30, KYSE-180, KYSE-150, KYSE-410, KYSE-510, and THP-1 were procured from Procell Life Science & Technology Co., Ltd. (Wuhan, China). TE-13 and TE-7 were obtained from FuHeng Biology Co., Ltd. (Shanghai, China) HET-1A and HEK293-FT were acquired from Fenghui Biotechnology Co., Ltd. (Hunan, China). The ESCC cell lines and HEK293-FT were grown in Dulbecco’s Modified Eagle Medium (DMEM) (Gibco, USA), whereas HET-1A was maintained in LHC-9 Medium (Gibco, USA), and THP-1 was sustained in RPMI-1640 (Gibco, USA). The DMEM media comprising with 10% FBS (NEWZERUM, New Zealand) and 1% PS (Gibco, USA). The growth medium for THP-1 cells included 0.05 mM β-mercaptoethanol. All cells were kept in a Heracell™ 150i CO_2_ Incubator (Thermo Scientific, Waltham, USA) at 37°C with 5% CO_2_.

### 2.3 Virus packaging and transfection

The ZC3H13 negative control plasmid (pReceiver-M68) (OE-NC), as well as the ZC3H13 shRNA interference vectors (shRNA1, 2, 3) and negative control plasmid (shRNA-NC), were procured from GeneCopoeia (Rockville, USA). Lentivirus packaging was conducted in HEK293-FT cells using HilyMax reagent (Dojindo, Japan). Stable cell lines with ZC3H13 knockdown (ZC3H13-shRNA) were established through co-culture of KYSE-150 and KYSE-410 cells with puromycin. ZC3H13 (NM_001076788.2) was cloned and inserted into pReceiver-M68 plasmid to construct ZC3H13 overexpression plasmid. After instantaneous transformation with HilyMax reagent, puromycin was performed for 2–3 months to obtain stable cell lines. The specific sequences for ZC3H13 and the shRNA interference are detailed in Appendix 1-1.

### 2.4 Macrophage polarization model

Following subculture and amplification, THP-1 cells were subjected to treatment with 100 ng/mL Phorbol 12-myristate 13-acetate (PMA) (Topscience, Shanghai, China) for 2 days in addition to the original medium, which led to the differentiation of THP-1 into M0 macrophages. Further polarization of M0 macrophages into M2 phenotype was induced by treating them with an additional 20 ng/mL IL-4 (Yeasen, Shanghai, China) and 20 ng/mL IL-13 (Yeasen, Shanghai, China) for 48 hours in the presence of PMA. Due to the adhesive nature of the induced macrophages and potential errors associated with flow cytometry, the polarization process of macrophages was assessed using IF and enzyme-linked immunosorbent assay (ELISA) in this study.

### 2.5 RNA extraction and qRT-PCR

The experiments were executed per the supplier’s protocols. Cells and tissues underwent RNA extraction employing Trizol (Invitrogen, Waltham, USA). The synthesis of complementary DNA (cDNA) was accomplished through the RevertAid™ First Strand cDNA Synthesis Kit, incorporating DNase I (Thermo Scientific, Waltham, USA). The quantitative real-time polymerase chain reaction (qRT-PCR) analysis was carried out utilizing PowerUp™ SYBR™ Green Mix (Applied Biosystems, Waltham, USA) in eight-tube strips and measured on a Bio-Rad CFX96 system (Bio-Rad, Hercules, USA). The results were evaluated using the 2^-ΔΔCT^method. The primer sequences utilized are detailed in Appendices 1-2.

### 2.6 Western blotting

Cell or tissue protein extraction utilized RIPA buffer for lysis. Protein samples underwent separation via 10% sodium dodecyl sulfate-polyacrylamide gel electrophoresis (SDS-PAGE) analysis. The separated proteins were subsequently transferred onto polyvinylidene difluoride (PVDF) membranes, succeeded by blocking using 5% skim milk powder (for nonphosphorylated proteins) or BSA (phosphorylated proteins), then incubated with primary antibodies at 4°C overnight. Horseradish peroxidase (HRP)-conjugated secondary antibodies were applied to the cells for 1 hour at 37°C. Protein bands were visualized using Enhanced Chemiluminescence (Biosharp, AnHui, China) and Tanon 5200 (Tanon, Shanghai, China). The antibodies used are detailed in Appendices 1-3.

### 2.7 ELISA

The ELISA experiment was executed per the supplier’s protocols. Raw data were procured utilizing MD SpectraMax Plus 384 (Molecular Devices, San Jose, USA). The following kits were employed for the study: Colorimetric m6A RNA Methylation Assay Kit (ab185912) (Abcam, Cambridge, England), Human RANTES ELISA Kit (CCL5) (ab174446) (Abcam, Cambridge, England), Human IL-8 ELISA Kit (ab214030) (Abcam, Cambridge, England), and the human Mannose Receptor (CD206) ELISA kit (ab277420) (Abcam, Cambridge, England).

### 2.8 Immunohistochemistry assays

Clinical ESCC pathological tissues and mouse cell line-derived xenograft (CDX) tumors underwent fixation in 4% paraformaldehyde, followed by paraffin embedding, sectioning, hydration, antigen retrieval, and endogenous peroxidase neutralization. The specimens were then treated with primary antibodies at 4°C overnight. After primary antibody treatment, the sections received HRP-conjugated secondary antibodies for 30 minutes at ambient temperature. Color visualization was achieved using DAB substrate, followed by hematoxylin counterstaining and dehydration prior to coverslip mounting. Two pathologists independently observed the pathological sections and confirmed the results. The antibodies utilized for IHC assays are detailed in Appendices 1-4.

### 2.9 EdU staining

The EdU labeling procedure utilized a BeyoClick™ EdU Cell Proliferation Kit containing Alexa Fluor 555 (Beyotime, Shanghai, China). The experimental cells underwent seeding in 24-well culture plates followed by EDU treatment. Following exposure to 37°C and 5% CO_2_ for 2 hours, the specimens underwent fixation with 4% paraformaldehyde and membrane permeabilization utilizing Triton X-100. Fluorescently labeled azide was added, and nuclear staining was performed using Hoechst 33342. Observations and recordings were made using a Nikon ECLIPS Ti-2 fluorescence microscope. Alexa Fluor 555. Ex: 555nm, Em: 565nm. Hoechst 33342. Ex: 346 nm, Em-460 nm.

### 2.10 Immunofluorescence assay

Cells were preconditioned onto cell slides, permeabilized through membranes, and blocked with 3% BSA. Single antibodies (mouse or rabbit) were used for single staining, while double antibodies from different sources (mouse and rabbit) were used for double staining. The specimens remained in a humidity chamber at 4°C for overnight incubation. Subsequently, appropriate fluorescence-conjugated secondary antibodies were introduced and maintained at ambient temperature for 60 minutes. Nuclei were counterstained with DAPI, and plates were sealed with an anti-fluorescence quench sealing agent. Fluorescence signals were collected using a laser confocal microscope OLYMPUS FV1000. DAPI. Ex: 330–380 nm, Em: 420 nm. 488. Ex: 465–495 nm, Em: 515–555 nm. CY5. Ex: 608–648 nm, Em: 672–712 nm.

### 2.11 Determination of cell viability

Cells were placed into 96-well cell culture plates and treated. The supernatant was discarded and replaced with a complete medium containing 10% CCK-8 (Dojindo, Japan). After a 1-hour incubation at 37°C in a 5% CO_2_ atmosphere, absorbance values of each well were measured at OD450 nm utilizing MD SpectraMax Plus 384 (Molecular Devices, USA).

### 2.12 Cell line-derived xenograft

A mixture containing 1×10^7^ cancer cells (KYSE-150) combined with VitroGel™3D ready-to-use hydrogel (Biological Industries, Haemek, Israel) underwent subcutaneous administration into the dorsal region of female BALB/c nude mice between 4–6 weeks of age. A mixture containing 10 mice were utilized, with 5 mice in each group. Following a four-week period, the mice were euthanized to harvest the tumor specimens. This investigation was sanctioned by the Ethics Committee of Sun Yat-sen University Cancer Center. Approval number: L102012023060E.

### 2.13 Migration and invasion assays (Transwell)

Cell migration and invasion were evaluated utilizing transwell chambers (Corning, Corning, USA). Unlike migration, invasion incorporates VitroGel™3D ready-to-use hydrogel into the chamber, and the rest of the procedure is the same. 5000 cells were added to 200µL of serum-free medium and transferred to transwell chambers, and 500 µL of cell complete medium containing 20% FBS was added to the well plates. The cells were incubated for 24 hours at 37°C in 5% CO_2_. Subsequently, the upper chamber was wiped clean with a cotton swab, washed once with Dulbecco’s Phosphate-Buffered Saline (DPBS), fixed with 4% paraformaldehyde, and stained with 0.1% crystal violet (Beyotime, Shanghai, China). Cell migration and invasion were observed under a microscope. Each experiment was performed in triplicate.

### 2.14 Colony formation assay

Following digestion, the cells were resuspended in medium, maintaining a cell density of 1000 cells per 2 mL, and then seeded in 6-well plates at 2 mL per well. Throughout this period, half of the medium was exchanged every 3 days, and the cultures were maintained in an incubator for 2 weeks. After incubation, the cells received one wash with DPBS, underwent fixation using 4% paraformaldehyde, and were subjected to 0.1% crystal violet staining. Subsequently, images of the stained cells were recorded utilizing a digital camera.

### 2.15 Experimental additives

In this study, RANTES/CCL5 Protein, h-CXCL8, SAH, and SX-682 were purchased from MedChemExpress (MCE, USA).

### 2.16 Transient transfection

For siRNA transfection, Lipofectamine™ RNAiMAX Transfection reagent (Invitrogen, Waltham, USA) was utilized. The transfection process strictly adhered to the kit instructions, and siRNA was obtained from RiboBio Co., Ltd. (Guangzhou, China) Synthesis procedures were followed, and the sequences are detailed in Appendices 1-6.

### 2.17 Statistical analysis

All experimental data were presented as mean ± standard deviation. Each experiment underwent a minimum of three independent replications. Statistical evaluation of the collected data utilized SPSS 22 and GraphPad Prism 9 software. Student’s t-test served to examine variations between groups, while Pearson’s test was implemented for correlation investigations. Statistical significance was established at p-value < 0.05.

## 3 Result

### 3.1 ZC3H13 was highly expressed in ESCC and correlated with m6A level and immune infiltration

**Figure 1:**
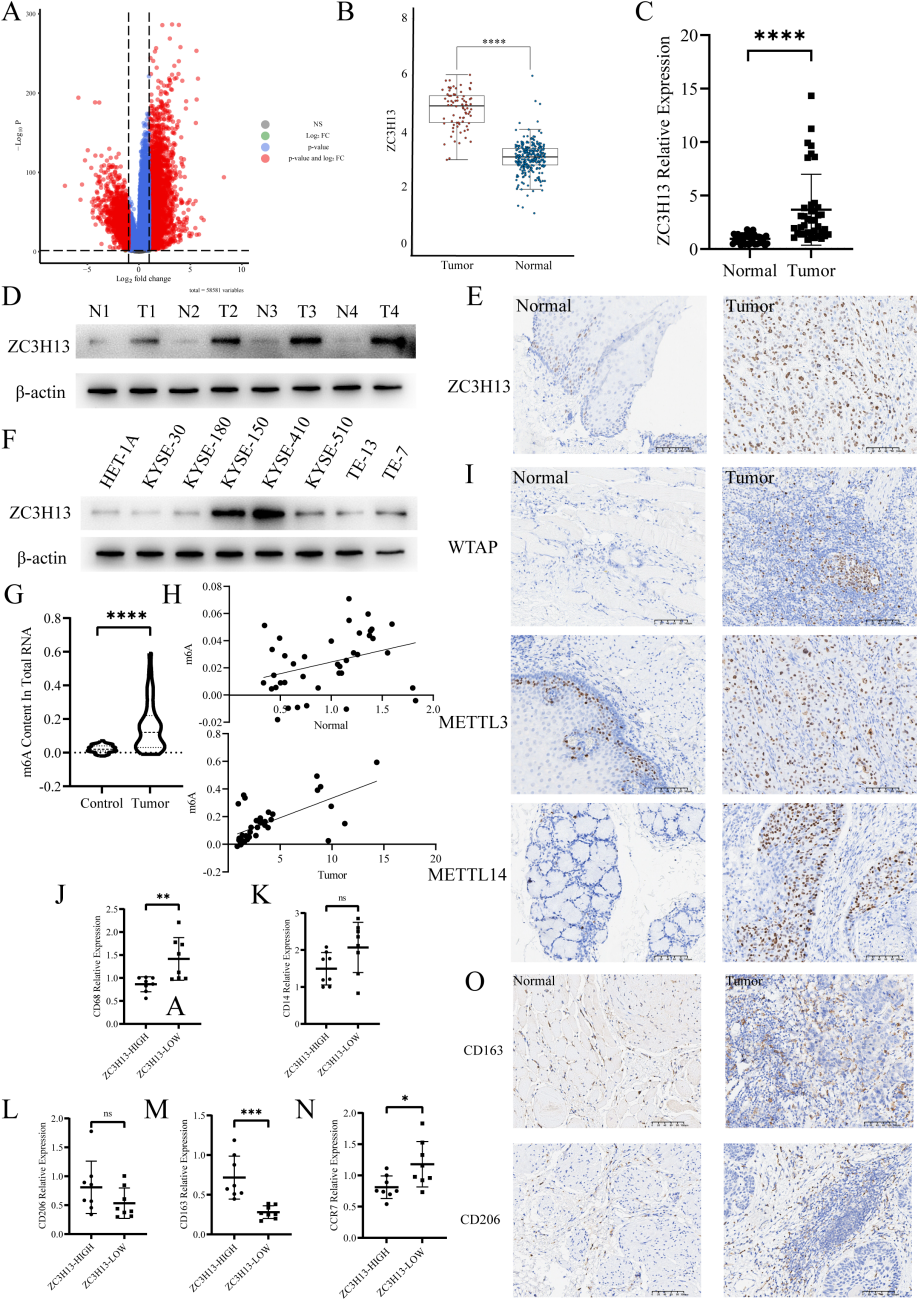
ZC3H13 expression is associated with a high m6A progression and M2 macrophage infiltration rate in ESCC tissues. **(A)** Volcano map. **(B)** TCGA database was used to analyze the expression of ZC3H13 in ESCC. **(C)** The relative expression of ZC3H13 in 40 pairs of ESCC tumor and non-tumor specimens was determined by qRT-PCR. **(D)** The relative expression of ZC3H13 in four pairs of ESCC tumor and non-tumor specimens was detected by WB. **(E)** Representative images of IHC staining showing ZC3H13 expression in ESCC tumor and non-tumor tissues (200×). **(F)** Expression of ZC3H13 in ESCC cells (WB). **(G)** Colorimetric method was used to measure m6a methylation in tissues. **(H)** Correlation between m6a methylation levels and ZC3H13 expression levels in tissues. Pearson correlation and two-tailed p values are shown. **(I)** IHC detection of WTAP, METTL14, and METTL3 expression and locus analysis in two groups with high and low ZC3H13 expression (200×). **(J–N)** Detection of CD68, CD14, CD206, CD163 and CCR7 expression by qPCR. **(O)** The expression of CD163 and CD206 in tissues was detected by IHC (200×). ns: not significant, *: p < 0.05, **: p < 0.01, ***: p < 0.001, ****:p < 0.0001.

**Figure 2:**
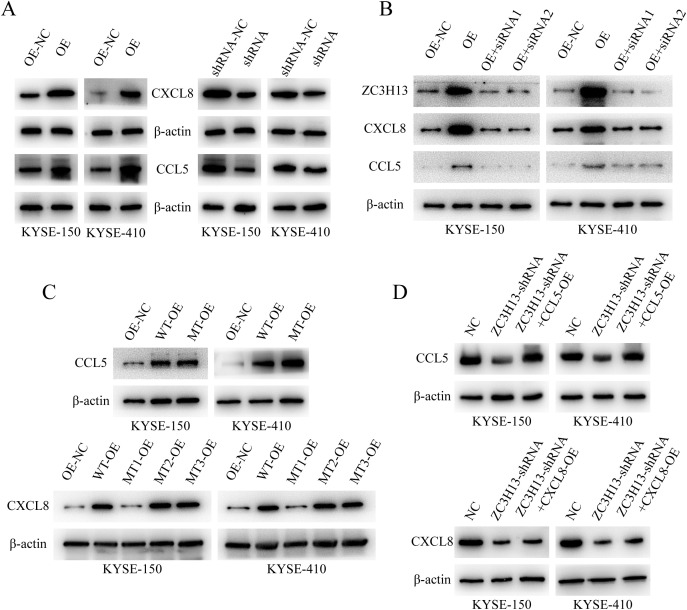
ZC3H13 regulates the expression of CCL5 and CXCL8. **(A)** The expression of CCL5 and CXCL8 in ZC3H13-OE-NC, ZC3H13-OE, ZC3H13-shRNA-NC and ZC3H13-shRNA cells was determined by WB. **(B)** WB was used to detect CCL5 and CXCL8 protein expression in ZC3H13-OE tumor cells and tumor cells that were interfered with ZC3H13 expression using siRNA. **(C)** The m6A modification site of CCL5 and CXCL8 was mutated, and the expression of CCL5 and CXCL8 was detected by WB. **(D)** In ZC3H13-shRNA, CCL5 and CXCL8 was overexpressed, and the expression of CCL5 and CXCL8 was detected by WB.

Initially, we examined the expression of ZC3H13 in the Genotype-Tissue Expression (GTEx) and The Cancer Genome Atlas (TCGA) dataset (**Figures 1A, B**). Differential expression analysis of genes associated with m6A methylation was conducted using the dataset (**Supplementary Figure S1A**). The qPCR results revealed a significant upregulation of ZC3H13 expression in ESCC samples (**Figure 1C**). This finding was further validated through Western blot (WB) analysis, demonstrating a marked elevate in ZC3H13 expression in ESCC samples versus normal esophageal tissues (**Figure 1D**). The quantitative analysis of Western Blot results for **Figures 1** and **2** is provided in the **Supplementary Material**. Immunohistochemical (IHC) staining of ESCC and adjacent normal tissues indicated that ZC3H13 was predominantly located in the nucleus and exhibited high expression in ESCC tissues (**Figure 1E**). We assessed ZC3H13 expression in ESCC cells through WB analysis, revealing upregulated expression in KYSE-150 and KYSE-410 compared to HET-1A (**Figure 1F**). Subsequently, the m6A methylation levels in ESCC and adjacent normal tissues were measured (**Figure 1G**). The m6A methylation level in ESCC tissues was significantly elevated and positively correlated with ZC3H13 expression (Pearson correlation coefficient (PCC): 0.646**). In contrast, there was a weaker correlation in adjacent normal tissues (PCC: 0.333*) (**Figure 1H**). ZC3H13’s association with m6A methylation in RNA modification was explored by performing IHC staining for m6A-related proteins WTAP, METTL14, and METTL3. The results showed that WTAP, METTL14, and METTL3 were all localized in the nucleus, with no significant correlation with the expression of ZC3H13 (**Figure 1I**). Using TCGA data, we analyzed the infiltration of 22 immune cell types in ESCC tumor tissues, revealing a significant upregulation of M2 macrophage infiltration (**Supplementary Figure S1B**). qPCR analysis of ESCC tissues with high and low ZC3H13 expression levels (ZC3H13-High and ZC3H13-Low) showed marked upregulation of CD163 expression and downregulation of CD68 and CCR7 in ZC3H13-High expression samples. The expression of CD206 and CD14 did not show significant differences between ZC3H13-High and ZC3H13-Low expression samples (**Figures 1–N**). IHC staining further confirmed that CD163 and CD206 signals were mainly concentrated in the peritumoral location (**Figure 1O**). These findings collectively demonstrate that ZC3H13 is highly expressed in ESCC and is linked to intracellular m6A modification levels and immune infiltration.

### 3.2 ZC3H13 promotes ESCC cell viability and xenograft tumor proliferation

**Figure 3:**
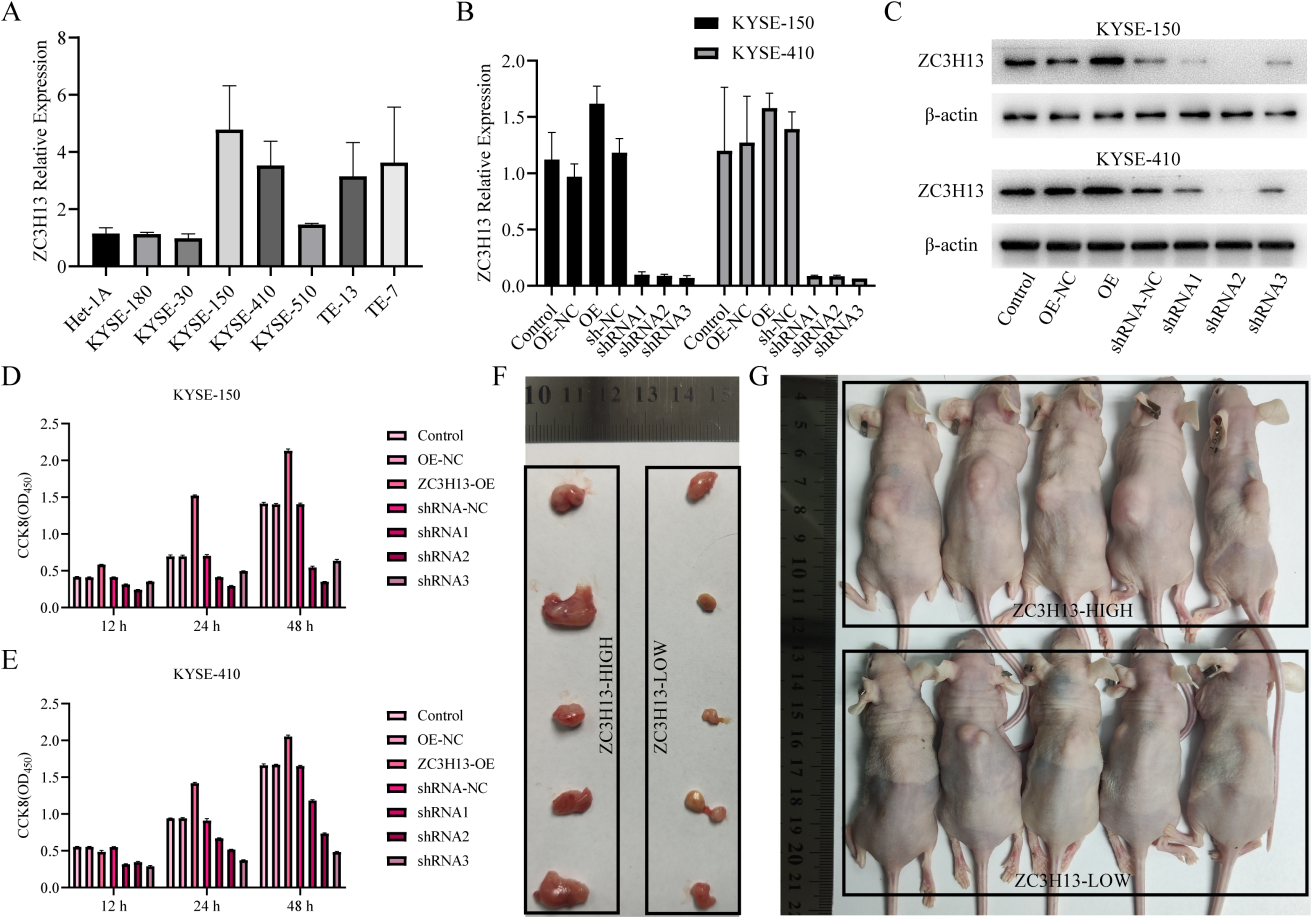
ZC3H13 promotes tumor proliferation *in vitro* and *in vivo* (KYSE-150). **(A)** Expression of ZC3H13 in ESCC cells (qPCR). **(B)** Construction of KYSE-150 and KYSE-180 of ZC3H13 stable transformants (qPCR). **(C)** Construction of KYSE-150 and KYSE-180 stably transformed ZC3H13 strains (WB). **(D, E)** CCK8 assay was used to detect the effect of ZC3H13 expression on ESCC cell viability. **(F)** *In vivo* tumor plots of CDX model mice with ZC3H13-shrNA-NC and ZC3H13-shRNA. **(G)** Panoramic images of CDX model mice.

We assessed ZC3H13 expression in ESCC cells through qPCR (**Figure 3A**) and WB (**Figure 1F**) analysis, revealing upregulated expression in KYSE-150 and KYSE-410 compared to HET-1A. Subsequently, qPCR and WB assays was used to detect ZC3H13 expression in stable cell lines with ZC3H13 overexpression and shRNA knockdown of KYSE-150 and KYSE-410 (**Figures 3B, C**). This resulted in the successful generation of ZC3H13 overexpression cell lines (ZC3H13-OE) and ZC3H13-shRNA interference cell lines (ZC3H13-shRNA). In subsequent experiments, shRNA2 were employed for KYSE-150 and KYSE-410 shRNA cell lines. CCK8 results showed that ZC3H13 overexpression had minimal correlation with cell viability, whereas downregulation of ZC3H13 inhibited cell viability (**Figures 3D, E**). To further elucidate the association between ZC3H13 and tumors, shRNA interference blank KYSE-150 cells (ZC3H13-shRNA-NC) and shRNA interference KYSE-150 cells (ZC3H13-shRNA) were subcutaneously injected into the back of nude mice (n = 5) (**Figures 3F, G**). Following a 28-day monitoring period, the mice were euthanized for tumor tissue collection. These findings collectively substantiate that ZC3H13 can exerting a role in maintaining or upregulating cell viability.

### 3.3 The expression of ZC3H13 is associated with m6A modification and immune infiltration in ESCC

**Figure 4:**
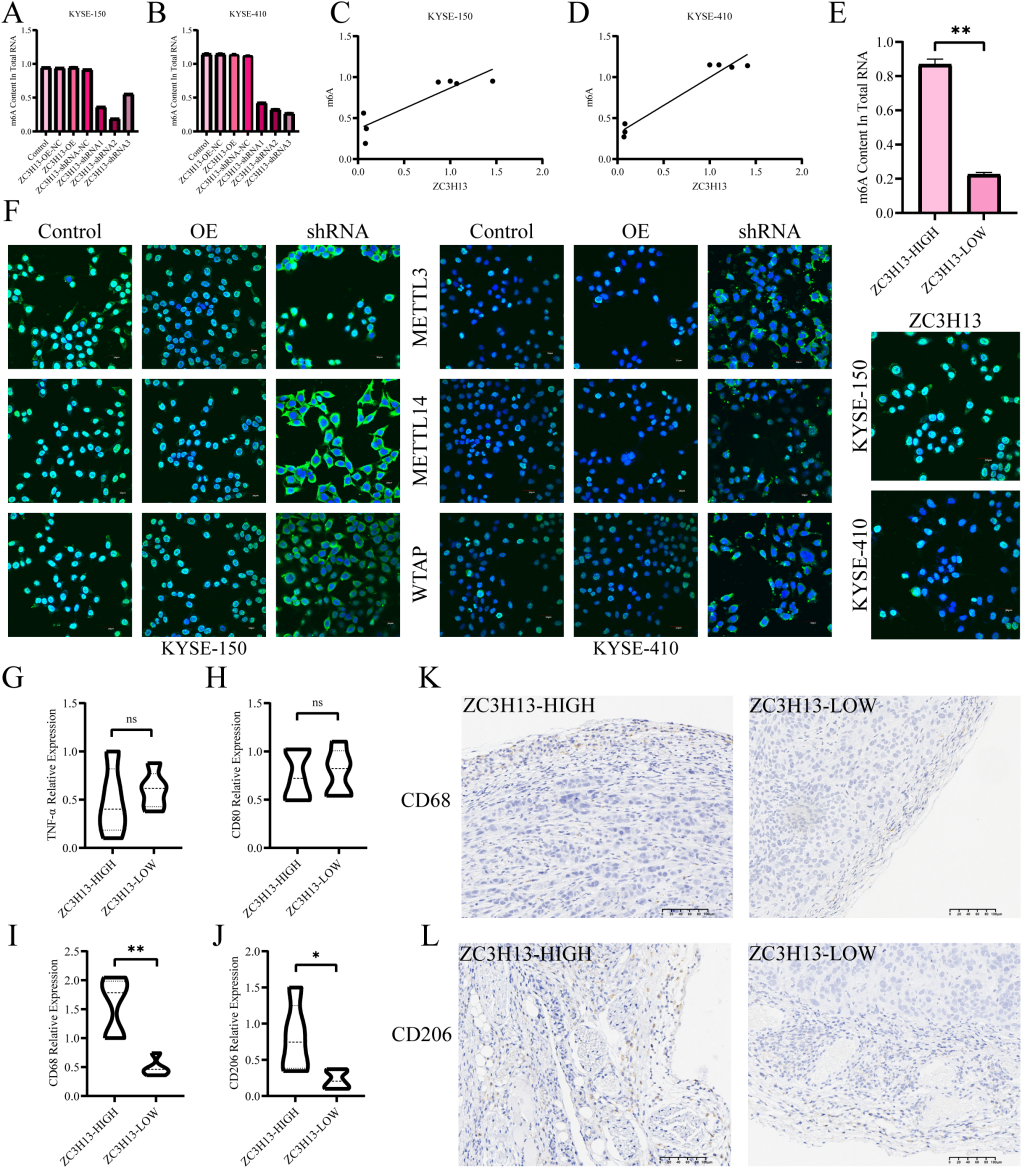
Association of ZC3H13 expression with m6A modification and immune infiltration in ESCC. **(A, B)** Colorimetric assay was used to measure intracellular m6a methylation. **(C, D)** Correlation between cellular ZC3H13 expression and m6a methylation levels. **(E)** Colorimetric method was used to detect m6a methylation in tissues. **(F)** IF staining was used to detect intracellular ZC3H13, WTAP, METTL14, and METTL3 (400×). **(G–J)** The relative expression of TNF-α, CD80, CD68 and CD206 in the ZC3H13-shrNA-NC (n = 5) and ZC3H13-shRNA (n = 5) groups was analyzed by qRT-PCR. **(K, L)** Representative images of CD68 & CD206 IHC staining in ZC3H13-shrNA-NC and ZC3H13-shRNA tumor tissues (200×). ns: not significant, *: p < 0.05, **: p < 0.01.

The m6A levels in ZC3H13-OE and ZC3H13-shRNA stably transfected cells were measured. Downregulation of ZC3H13 led to a significant decrease in intracellular m6A levels, while upregulation did not result in significant changes. There was a positive link between intracellular m6A levels and ZC3H13 expression (**Figures 4A–D**). Additionally, the m6A levels in tissues revealed that the downregulation of ZC3H13 led to the suppression of m6A modification in tumors (**Figure 4E**). Immunofluorescence (IF) staining indicated that ZC3H13 inhibition led to suppressed nuclear transport of WTAP, METTL14 and METTL3, with no notable variation between the ZC3H13 overexpression group and the control (**Figure 4F**). The infiltration of M2 macrophages was analyzed through real-time quantitative PCR (qRT-PCR) and IHC (**Figures 4G, H, K**). Notably, the ZC3H13-shRNA-NC group exhibited significantly higher infiltration of CD68+ macrophages compared to the ZC3H13-shRNA group, indicating that ZC3H13 promotes macrophage infiltration *in vivo*. To delineate macrophage subtypes, qRT-PCR and IHC assays were conducted (**Figures 4I, J, L**). The results highlighted that the heterogeneity in macrophage infiltration in tumor tissues induced by ZC3H13 predominantly involved CD206+ macrophages, indicative of M2 macrophages.

### 3.4 ZC3H13 mediated m6A modification of CXCL8 mRNA and CCL5 expression

WB analysis of CCL5 and CXCL8 expression in ESCC cells showed that when ZC3H13-shrna inhibited ZC3H13 expression, CCL5 and CXCL8 expression was down-regulated, and when ZC3H13-OE promoted ZC3H13 expression, CCL5 and CXCL8 expression was up-regulated (**Figure 2A**). Furthermore, silencing the overexpression of ZC3H13 with ZC3H13-siRNA reversed the upregulation of CCL5 and CXCL8 (**Figure 2B**). We next predicted m6A methylation sites for CXCL8 and CCL5 (**Supplementary Figures S2A, B**). CXCL8 mRNA was mutated at positions 5 (A to T), 183 (A to G), and 243 (A to G). The fifth base corresponded to the 5’-UTR of CXCL8 mRNA, while bases 183 and 243 corresponded to GAA. After amino acid codon optimization, GAA was replaced by GAG, both coding for glutamic acid. Similarly, experiments were conducted on predicted CCL5 by mutating base A of CCL5 at position 354 (A→T). PCR was performed through site-directed mutagenesis and then transfected into ESCC cells. WB results showed that the overexpression of CXCL8 failed to upregulate CXCL8 expression when the fifth base mutation was carried out, but other mutants or the wild type did, and no significant change in CCL5 expression due to the mutation (**Figure 2C**). In ZC3H13-shRNA cells, CXCL8 and CCL5 were overexpressed. When ZC3H13 expression was inhibited, the expression of CXCL8 and CCL5 was significantly downregulated. The overexpression of CXCL8 did not counter the downregulation caused by the inhibition of ZC3H13 expression. However, overexpression of CCL5 could compensate for the downregulation of CCL5 expression caused by inhibition of ZC3H13 expression (**Figure 2D**). The above findings suggest that ZC3H13 can regulate both CXCL8 and CCL5, although the underlying mechanisms may differ.

### 3.5 ZC3H13 drives M2 macrophage polarization through the CXCL8/CXCR2 axis

**Figure 5:**
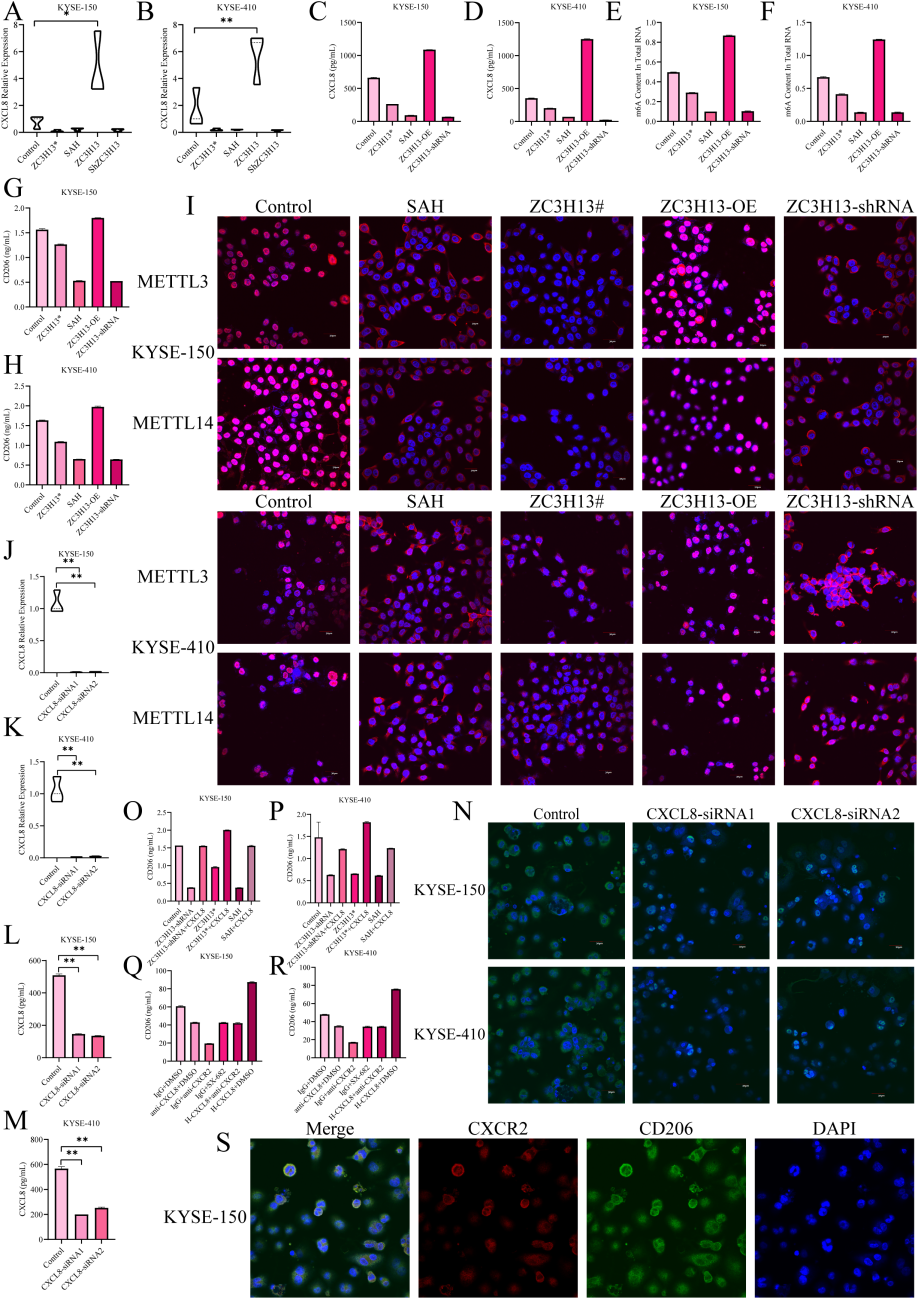
ZC3H13 drives M2 macrophage polarization via the CXCL8/CXCR2 axis. **(A, B)** The expression of CXCL8 was detected by qPCR. **(C, D)** CXCL8 expression in cell supernatant was measured by ELISA. **(E, F)** Colorimetric assay to measure intracellular m6a methylation. **(G, H)** Reduced M2 macrophage polarization was observed in conditioned media for both ZC3H13# and SAH cultures. **(I)** IF mapped the expression sites of WTAP, METTL14, and METTL3 (200×). **(J, K)** In KYSE-150 and KYSE-180 cell lines, CXCL8 was knocked down. **(L, M)** CXCL8 content in the culture medium supernatant was measured by ELISA. **(N)** After CXCL8 knockdown, reduced M2 macrophage polarization was observed in co-culture (400×). **(O, P)** M2 macrophage polarization was upregulated by the addition of recombinant h-CXCL8 to the culture medium. **(Q, R)** Reduced M2 macrophage polarization was observed by the addition of the CXCR2 inhibitor SX-682 or anti-CXCL8 antibody to the conditioned medium of ZC3H13 overexpression cultures. **(S)** Immunofluorescence staining showed that CXCR2 expression was co-localized with CD206 in the tumor, both on the membrane (400×). *: p < 0.05, **: p < 0.01.

Current evidence suggests that the structural integrity of ZC3H13 is crucial for the translocation of the WMM complex into the nucleus. In our investigations, we developed a ZC3H13 mutant, cloning the sequence 1–1460 of ZC3H13 and stably transfecting it into ESCC cells. Moreover, both ZC3H13# and SAH significantly downregulated the content of CXCL8 in the cell supernatant, mirroring the effect of direct ZC3H13 intervention (**Figures 5A–D**). Subsequent examination of the m6A modification level in cells revealed that ZC3H13# and SAH, similar to shRNA interference, downregulated the intracellular RNA m6A modification level (**Figures 5E, F**). Treatment with ZC3H13#, SAH (an inhibitor of METTL3-METTL14 heterodimer complex), or shRNA interference effectively inhibited the polarization of M0 to M2 macrophages (**Figures 5G, H**). Both ZC3H13# and SAH demonstrated the capacity to inhibit the nuclear translocation of METTL3 and METTL14 (**Figure 5I**). Knockdown of CXCL8 in KYSE-150 and KYSE-410 cells resulted in a substantial downregulation of CXCL8 in the cell supernatant (**Figures 5J–M**). The induction of M0 macrophage polarization was inhibited by adding an M2 macrophage induction reagent in the supernatant, as demonstrated by IF staining (**Figure 5N**). Additionally, the induction of M0 macrophages’ polarization by exogenous addition of h-CXCL8 in the supernatant reversed the inhibitory effects caused by shRNA interference, ZC3H13 mutation, and SAH treatment (**Figures 5O, P**). Interfering with CXCL8 activity in cell supernatant or inhibiting the macrophage CXCR2 receptor inhibited M2 macrophage polarization (**Figures 5Q, R**). IF co-staining of CXCR2 and CD206 expression sites in M2 macrophages revealed localization on the cell membrane in M2 macrophages (**Figure 5S**).

### 3.6 ZC3H13 regulates CCL5 expression and promotes M0 macrophage migration

**Figure 6:**
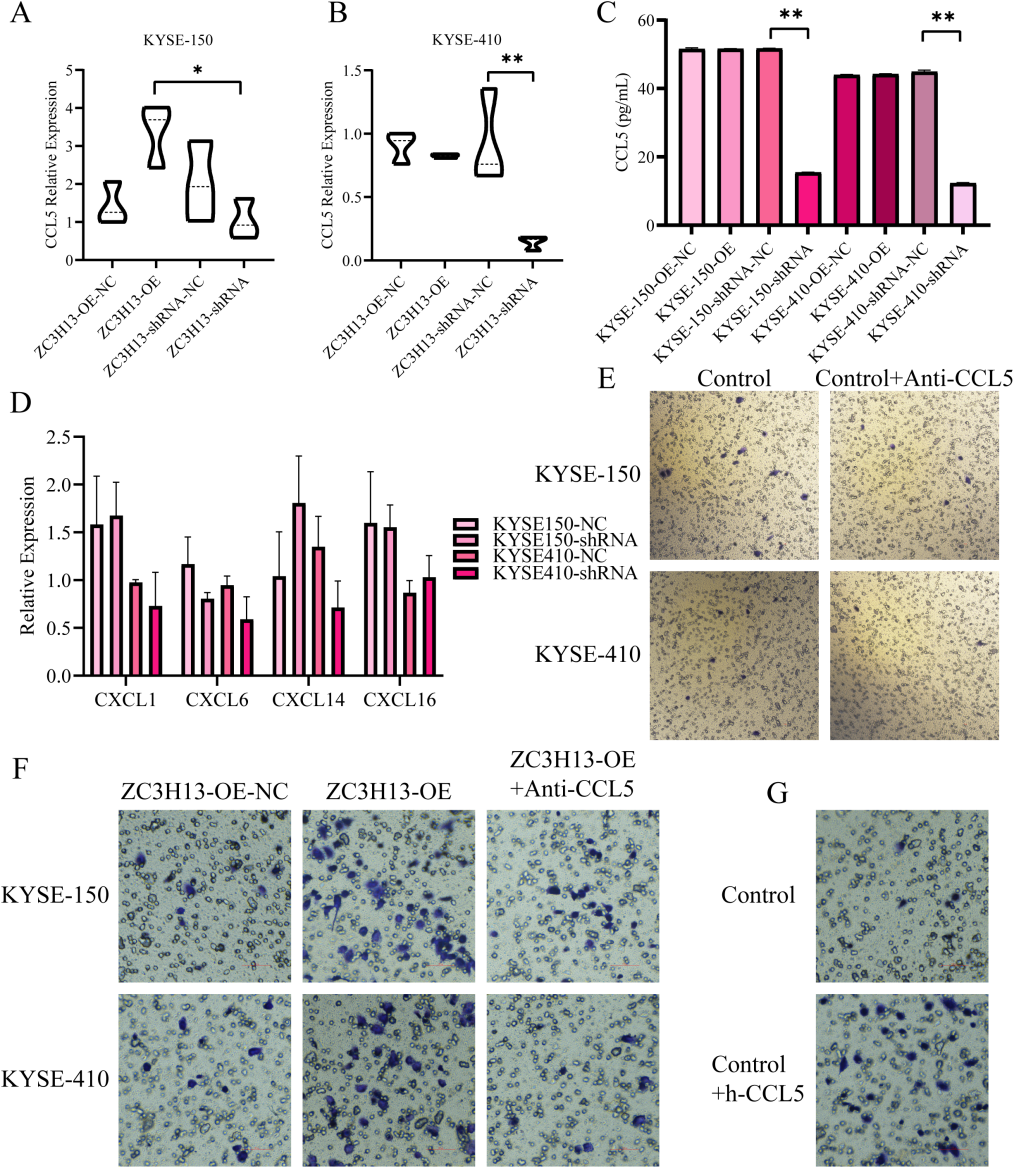
ZC3H13-positive tumor cells promote the recruitment of M0 macrophages by secreting CCL5. **(A, B)** The relative expression of CCL5 in ZC3H13-OE and ZC3H13-shRNA tumor cells was determined by qRT-PCR. **(C)** The relative expression of CCL5 in ZC3H13-OE and ZC3H13-shRNA tumor cells was measured by ELISA. **(D)** Relative expression levels of CXCL1, CXCL6, CXCL14, and CXCL16 in ZC3H13-OE and ZC3H13-shRNA tumor cells were determined by qRT-PCR. **(E)** CCL5 secretion by ESCC cells can induce the migration of M0 macrophages (200×). **(F)** Results of M2 macrophage migration assay induced by blocking ZC3H13-OE tumor cells with 1.0 µg/mL anti-CCL5 (200×). **(G)** Results of the M2 macrophage migration assay using serum-free medium and 10 ng/mL CCL5 protein, respectively (200×). *: p < 0.05, **: p < 0.01.

Given ZC3H13’s role in regulating the nuclear translocation of METTL3, an analysis of the Gene Expression Omnibus (GEO) dataset GSE179267 identified m6A methylation modifications on CCL5, CXCL1, CXCL6, CXCL8, CXCL14, and CXCL16. qPCR and ELISA results revealed significant downregulation of CCL5 expression in ESCC cells with ZC3H13 knockout (**Figures 6A–C**), while the expression of CXCL1, CXCL6, CXCL14, and CXCL16 showed minimal changes (**Figure 6D**). In a one-step method, THP-1 was induced into M0 macrophages, and a transwell migration assay was conducted with anti-CCL5 antibody added to neutralize CCL5 in the culture medium. The results showed that anti-CCL5 attenuated the pro-migration effect of M0 macrophages induced by ZC3H13 (**Figure 6E**). However, the effect on M2 macrophages was weak (**Figure 6F**). Subsequently, recombinant h-CCL5 was used to induce M0 macrophage migration, revealing that h-CCL5 significantly promoted the migration of M0 macrophages (**Figure 6G**). Taken together, these findings indicate that ZC3H13-mediated CCL5 could promote M0 macrophage migration.

### 3.7 M2 macrophages promote ESCC proliferation, migration and invasion

**Figure 7:**
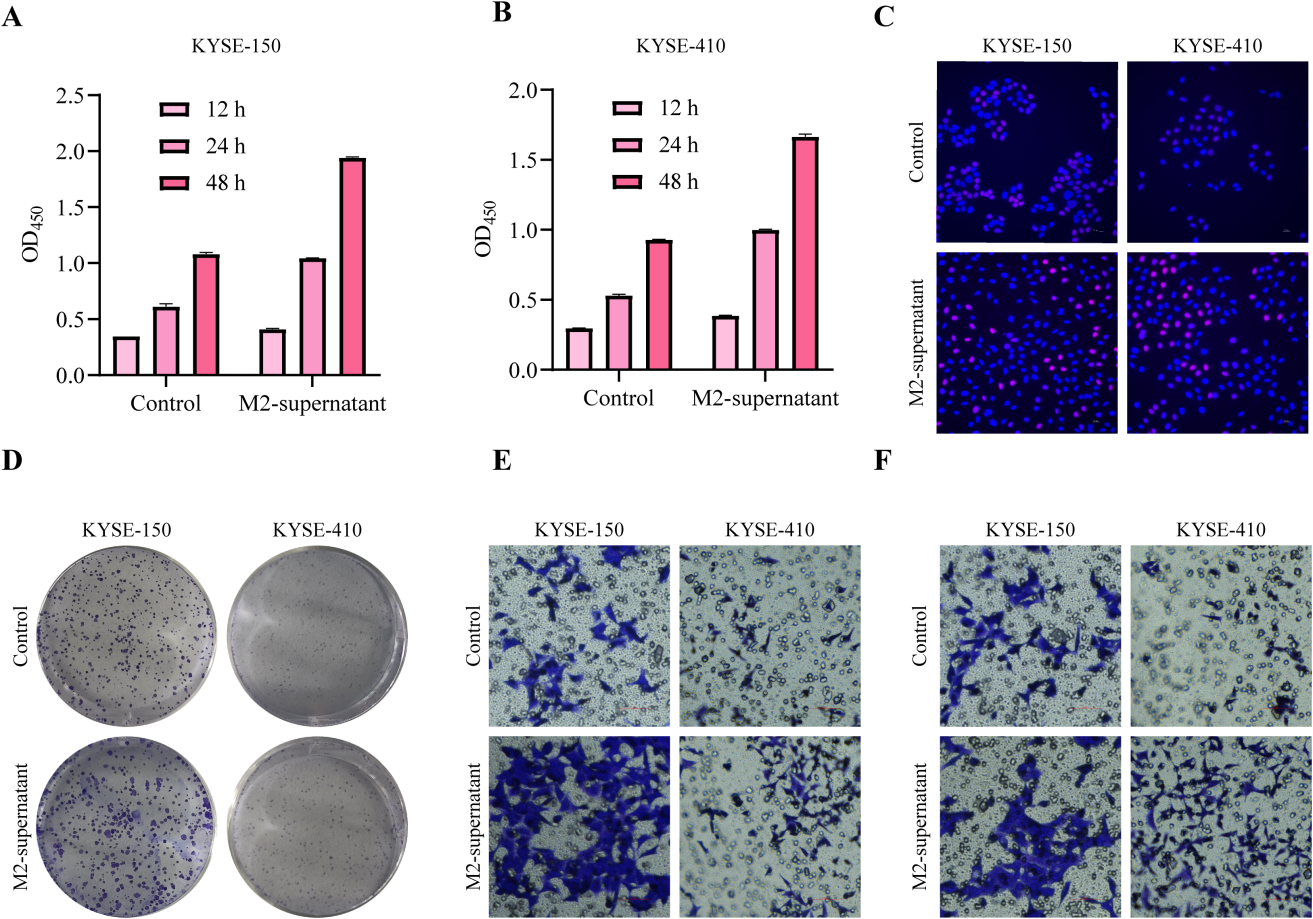
M2 macrophages promote tumor cell proliferation and migration. **(A, B)** After treatment with M2-supernatant or control medium, the cell viability of tumor cells was measured by CCK-8 assay. **(C)** EdU staining was used to visualize the proliferative capacity of tumor cells treated with an M2 supernatant or control medium (200×). **(D)** After treatment with an M2-supernatant or control medium, the proliferation ability of tumor cells was determined by clone formation assay. **(E)** Transwell migration assay treated with M2-supernatant or control medium (200×). **(F)** Transwell invasion assay treated with M2-supernatant or control medium (200×).

M2 macrophages, known for secreting various tumor-promoting factors and extracellular vesicles (EVs), serve a pivotal function in tumor occurrence and development. To investigate the influence of M2 macrophage secretion on tumor cells, we induced M0 macrophages to undergo M2 macrophage polarization, cultured M2 macrophages in a serum-free medium, collected M2 supernatant, and treated ESCC tumor cells. Functional analysis demonstrated that ESCC cells exhibited higher cell viability when cultured in M2 supernatant (**Figures 7A, B**). 5-Ethynyl-2’-deoxyuridine (EdU) staining showed that M2-supernatant upregulated cell proliferation activity (**Figure 7C**). The clone formation assay revealed that M2 supernatant enhanced the ability of tumor cells to form colonies (**Figure 7D**). Transwell assays demonstrated that M2 supernatant promoted the migration (**Figure 7E**) and invasion (**Figure 7F**) of ESCC cells. Based on these findings, it can be inferred that M2 supernatant promotes the proliferation, migration, and invasion of ESCC cells.

## 4 Discussion

ZC3H13, as a pivotal m6A methyltransferase within the m6A-MeTTL-associated complex (MACOM), holds a crucial role in the m6A methylation process and is instrumental in the nuclear translocation of m6A methyltransferase (17). Our study findings were consistent with previous findings in mouse embryonic stem cells, where ZC3H13 knockdown resulted in the inhibition of nuclear translocation of WTAP, Virilizer, and Hakai (18). Herein, the irreversible impact on the nuclear translocation of WTAP, METTL3, and METTL14 was observed upon knocking out or mutating ZC3H13. Although MeRIP-seq is conventionally employed for m6A methylation experiments, we leveraged the GEO database to assess the results of METTL3 knockdown in ESCC cells. Analysis of the GSE179267 (19) dataset revealed consistent findings, confirming the regulation of CCL5 and CXCL8 by ZC3H13. Importantly, the modulation of CXCL8 may be associated with m6A modification, while the regulation of CCL5 might be linked to downstream signaling pathways regulated by ZC3H13.

CCL5, a member of the CC chemokine family, functions in inducing cell chemotaxis (20). Previous studies, particularly in hepatocellular carcinoma (HCC), have demonstrated the inhibition of M2 polarization of THP-1 M0 macrophages through the CCL5-CCR5 signaling pathway using Maraviroc (MVC), mediated by the STAT3-SOCS3 signaling pathway (21). Chordoma-induced secretion of CCL5 has been linked to macrophage recruitment and M2 polarization, with MVC showing inhibitory effects on these processes and subsequently suppressing chordoma proliferation, migration, and invasion (22). Another study revealed that the overexpression of 12-LOX in ESCC, induced by radiotherapy, upregulates CCL5 expression, promoting THP-1 induction into macrophages and M2 polarization. This, in turn, enhances the migration ability of ESCC cells (23). In prostate cancer (PCa), ERα positivity inhibits CCL5 expression in cancer-associated fibroblasts (CAFs), leading to reduced macrophage infiltration, migration of M2 macrophages, and inhibition of prostate cancer invasion (24). Furthermore, induction of M0, M1, and M2 macrophages with CCL2, CCL5, CXCL10, CXCL12, and C1q revealed that M2 macrophages exhibited the longest migration distance compared to M0 and M1 macrophages (25). MLN4924 was found to promote the infiltration of M2 macrophages by mediating CCL5, enhancing pancreatitis activity. Blocking CCL5 or clearing macrophages was effective in reducing MLN4924-enhanced pancreatitis and fibrosis (26). Consistently, we found that ZC3H13 could promote CCL5 expression and M2 macrophage migration in ESCC.

CXCR2, a G Protein-Coupled Receptor (GPCR) within the chemokine receptor family (27), interacts with ligands such as CXCL1, CXCL2, CXCL3, CXCL5, CXCL6, CXCL7, and CXCL8 (28). In HCC, despite the inability of CXCR2 knockout to inhibit tumor growth, it was found to promote M1 macrophage polarization in tumors and reduce PD-L1 expression through the mediation of c-Myc down-regulation (29). M2-TAMs in gastrointestinal stromal tumors (GIST) were observed to secrete CXCL2, enhancing the migration, invasion, and EMT of GIST cells via the CXCL2-CXCR2 signaling pathway (30). Pten-null prostate tumors were noted to promote TAM infiltration and polarization to an anti-inflammatory phenotype (M2 polarization) through the CXCL2-CXCR2 signaling pathway. Blocking CXCR2 showed promise in restoring TAM to a pro-inflammatory phenotype (31). The combination of anti-CXCL1-CXCR2 blockade with doxorubicin in HCC treatment was found to reduce TAM recruitment in the tumor microenvironment (TME) and inhibit tumor progression (32). Herein, we successfully inhibited the M2 polarization of TAMs by blocking CXCL8-CXCR2, although it should be borne in mind that our experiments were limited to the cellular level, lacking animal experiments for further validation.

CXCL8, also known as interleukin-8 (IL-8), is a member of the CXC chemokine family, acting as an agonist for CXCR1/CXCR2 (33). In the context of pancreatic cancer (PC), macrophages with a CXCR2 phenotype display immunosuppression due to M2 polarization. Combining PD1 blockade therapy with the inhibition of tumor-secreted CXCL8 and CXCR2-positive M2 macrophages enhances therapeutic efficacy (34). ESCC demonstrates significantly upregulated expression of tryptophan 2,3-dioxygnease 2 (TDO2), inducing M2 macrophage polarization through the activation of the AKT/GSK3 signaling pathway and upregulation of IL-1β (35). Gastric cancer-derived mesenchymal stromal cells (GC-MSCs) secrete high levels of IL-6 and IL-8 in the TME, leading to M2 macrophage polarization, promoting EMT, and facilitating gastric cancer metastasis (36). Another study revealed that IRGM promotes glioma development by regulating the p62/TRAF6/NF-κB pathway, resulting in increased IL-8 production and M2 macrophage polarization (37). The co-expression of NTS and IL-8 in HCC indicates that IL-8 induces M2-type TAMs in the HCC TME, indirectly promoting EMT (38). Bladder cancer (BCa) exhibits SULF2-mediated IL-8 secretion through β-catenin, inducing M2 macrophage polarization via the JAK2/STAT3 signaling pathway (39). In our study, ZC3H13 was identified as a key player in the m6A modification of CXCL8 mRNA, resulting in the upregulation of CXCL8 secretion in ESCC.

## 5 Conclusion

**Figure 8:**
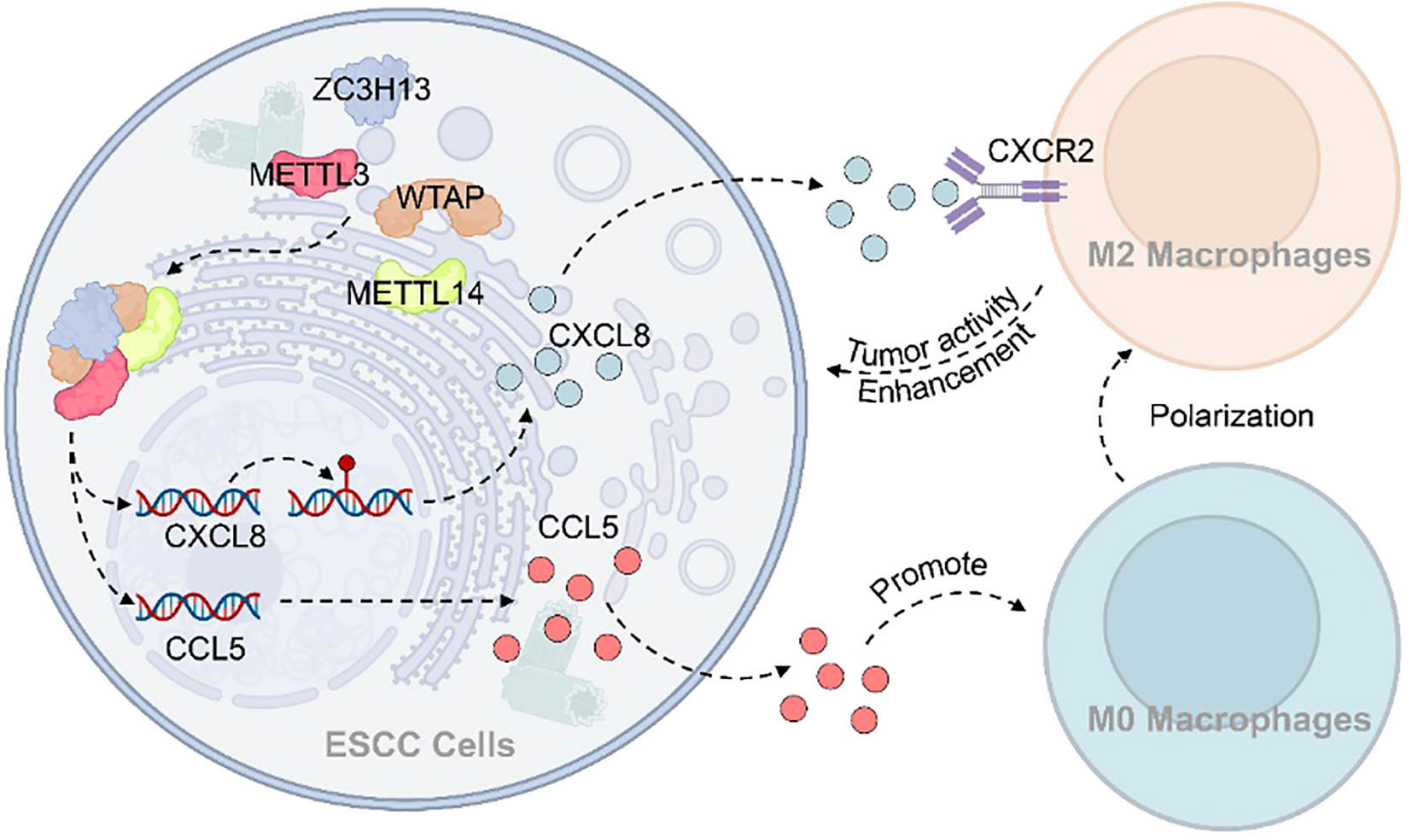
Mechanism diagram of ZC3H13 regulating the immune microenvironment of ESCC.

There is growing evidence supporting the tumor-promoting role of M2 tumor-associated macrophages (M2-TAMs) within the tumor microenvironment (TME), highlighting their potential as promising therapeutic targets in cancer treatment. Mechanistically, this study demonstrates that ZC3H13 facilitates the nuclear translocation of WTAP, METTL3, and METTL14, thereby enhancing m6A methylation of CXCL8. This modification promotes the expression and secretion of CXCL8 and CCL5 in ESCC, which in turn drives the recruitment and polarization of M2 macrophages. The resulting shift in the immune microenvironment suppresses anti-tumor immune responses and ultimately supports tumor progression (**Figure 8**). Collectively, these findings advance our understanding of the complex interplay between m6A methylation, chemokine signaling, and immune regulation in esophageal squamous cell carcinoma, offering potential avenues for future therapeutic intervention.

## Data Availability

The original contributions presented in the study are included in the article/**Supplementary Material**. Further inquiries can be directed to the corresponding authors.
