# Supplementary material for: The role of ZC3H13 in promoting M2 macrophage infiltration via m6A methylation in esophageal squamous cell carcinoma tumor progression
Source: Front Immunol. 2025 Sep 1;16:1612041. doi: 10.3389/fimmu.2025.1612041 (PMC12434101; doi:10.3389/fimmu.2025.1612041)
Supplement: Supplementary file 1 [file Table1.docx]

1-1 The sequence number of ZC3H13 and the sequence of the shRNA interference targets

| ZC3H13 | NM_001076788.2 |
| --- | --- |
| ZC3H13-shRNA1 | GGATTTATGTCTGGATCATCC |
| ZC3H13-shRNA2 | GCTGGCGTGGTATGTTTATAG |
| ZC3H13-shRNA3 | GCAGACAGTAGTAGTGATAAT |

1-2 Primers were used for the qPCR

| CD163 | F: GGGATGTCCAACTGCTATCAA |
| --- | --- |
| NM_004244 | R: GACTCATTCCCACGACAAGAA |
| CD206 | F: GGACGTGGCTGTGGATAAAT |
| NM_002438 | R: ACCCAGAAGACGCATGTAAAG |
| ZC3H13 | F: CGGACACTAACTCCACCTTTAC |
| NM_001076788.2 | R: GTCTTGGAGGACTCTGCTTTC |
| CD68 | F: ACGCAACTGGCTCAAAGA |
| NM_001251.3 | R: TCCCAAAGTGCTGGGATTAC |
| CCR7 | F: GGTCGTGGTCTTCATAGTCTTC |
| NM_001838.4 | R: CAGGTGCTACTGGTGATGTT |
| CD14 | F: CTCAGAGGTTCGGAAGACTTATC |
| NM_000591.4 | R: TTCATCGTCCAGCTCACAAG |
| CCL5 | F: TGCCCACATCAAGGAGTATTT |
| NM_002985.3 | R: GATGTACTCCCGAACCCATTT |
| CXCL8 | F: CTTGGCAGCCTTCCTGATTT |
| NM_000584.4 | R: GGGTGGAAAGGTTTGGAGTATG |
| CD68(mouse) | F: ATTGAGGAAGGAACTGGTGTAG |
| NM_001291058.1 | R: CCTCTGTTCCTTGGGCTATAAG |
| CD206(mouse) | F: GGAATCAAGGGCACAGAGTTA |
| NM_008625.2 | R: TTCCATCTGCTCCACAATCC |

1-3 WB used the antibody

| Anti-ZC3H13 antibody | Abcam | ab138150 |
| --- | --- | --- |
| Anti-RANTES (CCL5) antibody | Abcam | ab307712 |
| Anti-IL-8 (CXCL8) antibody | Abcam | ab289967 |
| Anti-beta Actin antibody | Abcam | ab8227 |
| Goat Anti-Rabbit IgG H&L (HRP) | Abcam | ab6721 |

1-4 IHC used the antibody

| Anti-ZC3H13 antibody | Abcam | ab138150 |
| --- | --- | --- |
| Anti-WTAP antibody | Abcam | ab195380 |
| METTL14 Rabbit mAb | Cell Signaling Technology | 48699 |
| Anti-METTL3 antibody | Abcam | ab195352 |
| Anti-CD163 antibody | Abcam | ab182422 |
| Anti-Mannose Receptor (CD206) antibody | Abcam | ab64693 |
| Anti-CD68 antibody | Abcam | ab303565 |
| Anti-CD80 antibody | Abcam | ab254579 |

1-5 IF used the antibody

| Anti-ZC3H13 antibody produced in rabbit | Sigma | HPA040140 |
| --- | --- | --- |
| Anti-WTAP antibody | Abcam | ab195380 |
| METTL14 Rabbit mAb | Cell Signaling Technology | 48699 |
| Anti-METTL3 antibody | Abcam | ab195352 |
| CD206 (MMR) Monoclonal Antibody | Invitrogen | MA5-28581 |
| Anti-IL-8Rβ antibody produced in rabbit | Sigma | SAB4501544 |
| Goat Anti-Rabbit IgG H&L (Cy5^®^) preadsorbed | Abcam | ab97077 |
| Goat Anti-Mouse IgG H&L (Alexa Fluor® 488) | Abcam | ab150113 |

1-6 siRNA sequence

| ZC3H13-siRNA1 | UAUAAUUGCUGCUAUAACCUU |
| --- | --- |
| ZC3H13-siRNA2 | UUGUUCUUCAUUCUUUCUCUA |
