# Supplementary material for: The role of ZC3H13 in promoting M2 macrophage infiltration via m6A methylation in esophageal squamous cell carcinoma tumor progression
Source: Front Immunol. 2025 Sep 1;16:1612041. doi: 10.3389/fimmu.2025.1612041 (PMC12434101; doi:10.3389/fimmu.2025.1612041)
Supplement: Supplementary file 2 [file Table2.docx]

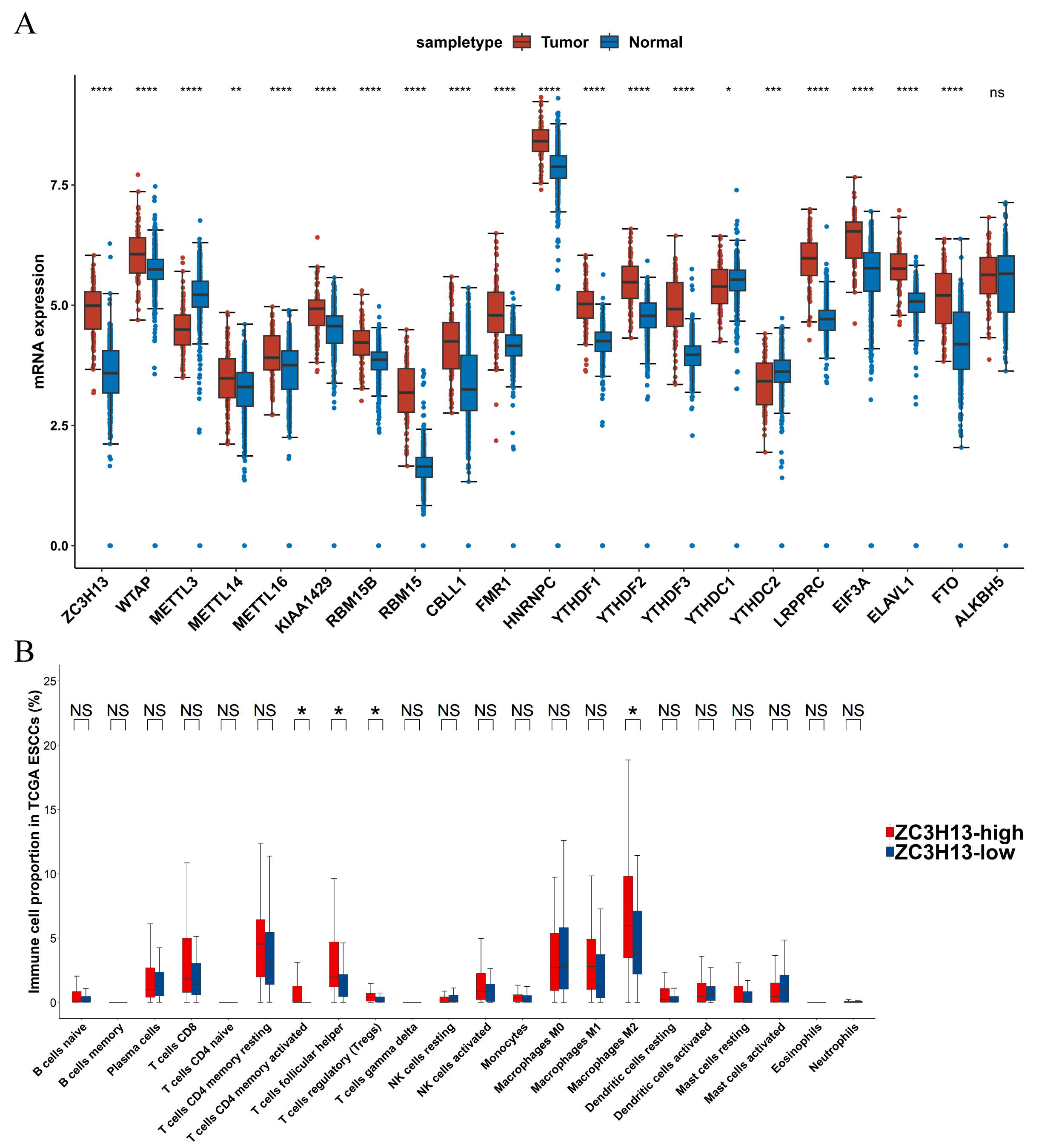


**FIG. S1**. Bioinformatics analysis was used to analyze the expression of m6A related genes and immune infiltration in tumors. A. Box plots of the m6A gene. B. Box plots of 22 immune cells infiltrating ESCC tumor tissues were analyzed in TCGA database.


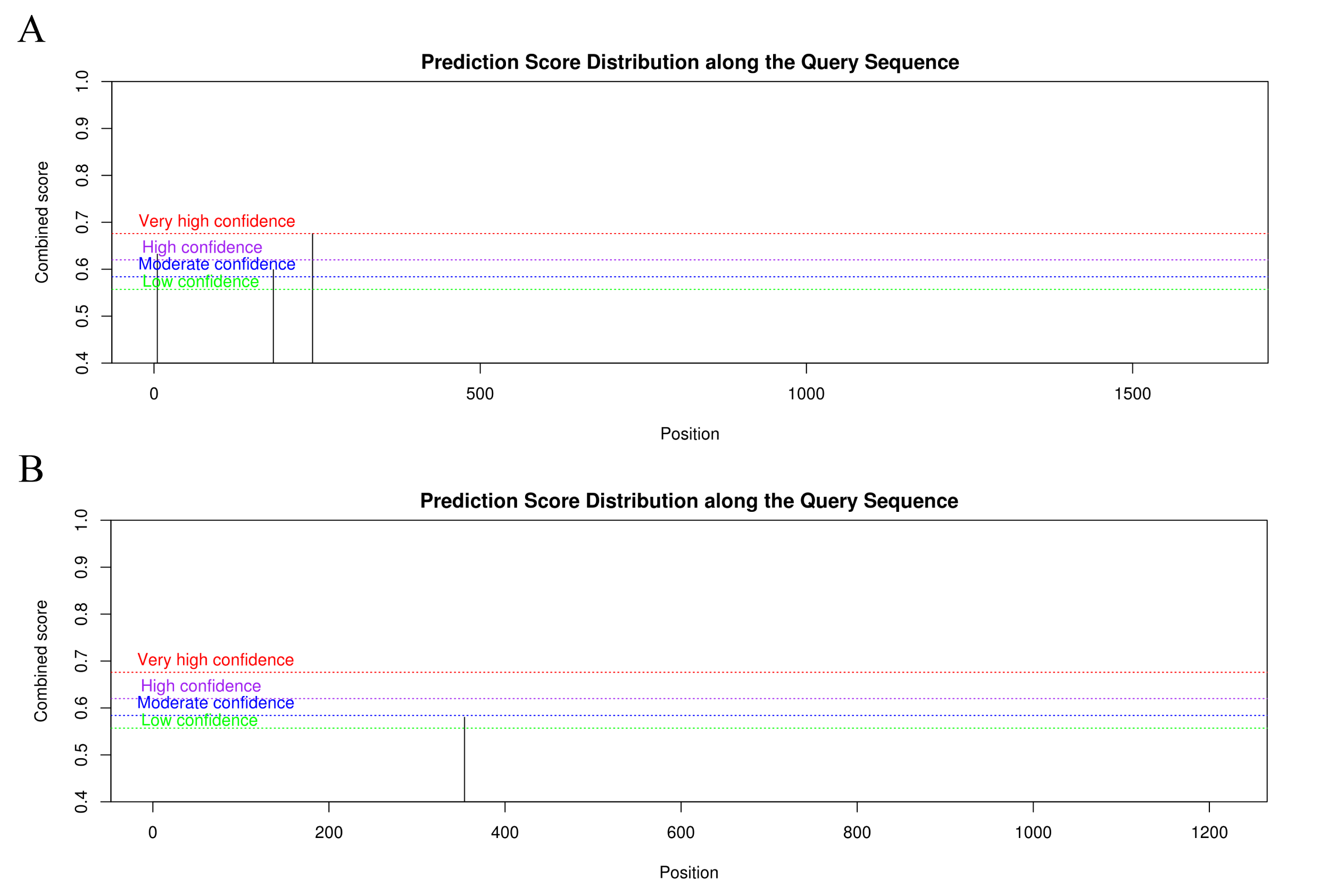


**FIG. S2**. Bioinformatics analysis was used to analyze the m6A methylation sites of CCL5 and CXCL8. A. SRAMP (http://www.cuilab.cn/sramp/) to predict CXCL8 m6A methylation site. B. SRAMP to predict CCL5 m6A methylation site.
